# Surface Hardening of Zr-1.0Sn-1.0Nb-0.3Fe Alloy Induced by Laser Surface Remelting

**DOI:** 10.3390/ma18173948

**Published:** 2025-08-22

**Authors:** Zhien Ning, Fangli Zhang, Lu Wu, Wei Zhang, Jijun Yang, Xiaotong Zhao, Linjiang Chai

**Affiliations:** 1 Key Laboratory of Radiation Physics and Technology of Ministry of Education, Institute of Nuclear Science and Technology, Sichuan University, Chengdu 610064, China; np201006@163.com (Z.N.); jjyang@scu.edu.cn (J.Y.); 2 The First Sub-Institute, Nuclear Power Institute of China, Chengdu 610041, China; 3 College of Materials Science and Engineering, Chongqing University of Technology, Chongqing 400054, China; zhangfangli@2020.cqut.edu.cn (F.Z.); zhaoxiaotong@stu.cqut.edu.cn (X.Z.); 4 State Key Laboratory of Advanced Nuclear Energy Technology, Nuclear Power Institute of China, Chengdu 610041, China; g_zlenovo@163.com

**Keywords:** zirconium alloy, laser surface remelting, hardness, microstructure, texture

## Abstract

To enhance surface hardness, laser surface remelting (LSR) was performed to treat the surface of a novel nuclear-grade Zr-1.0Sn-1.0Nb-0.3Fe zirconium alloy. A combination of advanced characterization techniques was used to systematically analyze the microstructural features of the samples before and after the LSR treatment, and their correlation with hardness variations was studied. Results show that the LSR-treated surface consists of two distinct microstructural regions: (*i*) the remelted zone (RZ), characterized by fine lath structures and precipitates distributed along the lath boundaries; and (*ii*) the heat-affected zone, comprising blocky α phase, α laths, and precipitates. The surface of the LSR-treated samples exhibits a random texture, which is attributed to the selection suppression of α variants during the laser-induced rapid transformation. The average hardness of the RZ reaches 285.7 ± 8.3 HV, ~40% higher than the substrate. This hardness enhancement is ascribed to LSR-induced grain refinement.

## 1. Introduction

Nuclear energy is widely recognized as one of the cleanest, most efficient, and economically viable energy sources. However, nuclear safety remains an essential foundation for the effective deployment of nuclear technology. Within the reactor core, materials face extremely harsh conditions, and the fuel cladding stands out as one of the most vital components for maintaining fuel integrity and ensuring the overall safety of nuclear power plants [1,2,3]. Because of their low thermal neutron absorption cross-section, outstanding corrosion resistance, and favorable mechanical properties, zirconium alloys have become the primary materials in the nuclear industry, extensively employed in manufacturing pressure tubes and fuel claddings for fission reactors [4]. After the 2011 Fukushima nuclear accident in Japan, the safety of fuel cladding drew heightened attention, leading to more stringent performance demands for cladding materials [5,6]. In the wake of the accident, developing advanced accident-tolerant fuel (ATF) claddings has become a globally recognized focus in nuclear materials research. Researchers from the United States, France, South Korea, and other countries have identified surface modification of zirconium alloy claddings as a promising short-term technical route within the broader ATF framework and have undertaken extensive investigations in this area [7].

A variety of surface modification techniques have been investigated to enhance the performance of zirconium alloys [6,8,9,10,11,12,13]. Among them, laser surface remelting (LSR) has garnered growing interest in recent years, owing to its benefits such as high processing efficiency, relatively low cost, and precise parameter control [14,15,16,17]. Previous studies have demonstrated that appropriate LSR treatment can significantly improve the surface properties of some zirconium alloys [18,19,20,21,22]. Compared with other surface modification methods, LSR employs a high-energy-density laser to instantaneously heat or melt the material surface, followed by rapid solidification, leading to the creation of a modified surface layer without altering the bulk chemical composition. This process induces substantial microstructural changes that can enhance surface performance (hardness, wear and corrosion resistance, etc.) while maintaining the inherent low thermal neutron absorption of zirconium alloys. Despite these advantages, the application of LSR for the surface modification of reactor-grade zirconium alloys remains relatively underexplored.

In this study, a novel nuclear-grade zirconium alloy recently developed in China was selected as the substrate material. A surface-modified layer was prepared using pulsed LSR, and the effects of this treatment on surface hardness were analyzed. In addition, multiple characterization techniques were jointly employed to investigate the effects of LSR on the microstructure and mechanical performance (hardness) of the alloy surface. The findings provide experimental evidence supporting the enhancement of in-reactor safety and reliability of zirconium alloy components, offering both academic value and engineering relevance.

## 2. Experimental Procedures

### 2.1. Materials

The starting material used in this study was a rolled and annealed zirconium alloy sheet, whose composition is provided in Table 1. Samples were sectioned from the as-received alloy sheet by a homemade wire cutting machine. Each sample measured 15 mm in length, 10 mm in width, and 2 mm in thickness, corresponding to the rolling (RD), transverse (TD), and normal (ND) directions, respectively.

### 2.2. Laser Surface Treatment

LSR was conducted on the surface (RD-TD) of the as-cut samples using an Nd:YAG pulsed laser system (Shenzhen United Winners Laser Co., Ltd., Shenzhen, China) with a 50% overlap rate. After comprehensive process optimization, the employed laser processing parameters are summarized in Table 2. During laser processing, argon gas was always blown (10 L·min^−1^) to protect the sample surface from oxidation. Argon gas was continuously supplied at a flow rate of 10 L·min^−1^ to protect the surface from oxidation during laser processing.

### 2.3. Microstructural Characterization

By using a PANalytical Empyrean Series 2 X-ray diffractometer (XRD; Malvern Panalytical B.V., Almelo, The Netherlands) equipped with Cu Kα radiation, phase analysis of both the as-received and LSR-treated samples was conducted. Detailed microstructural characterization of the cross-sectional features was then carried out using a Zeiss Sigma HD field emission scanning electron microscope (Carl Zeiss Microscopy GmbH, Jena, Germany) with an electron backscatter diffraction (EBSD) system and an energy-dispersive X-ray spectroscope (EDS). Before analysis, the sample surfaces were sequentially ground with abrasive papers ranging from 180 to 5000 grit, followed by electrolytic polishing at 20 V and −30 °C for 40 s in an electrolyte composed of a 9:1 (vol.%) mixture of anhydrous ethanol and perchloric acid.

### 2.4. Hardness Testing

Prior to hardness testing, the cross-sectional surfaces of the samples were ground with abrasive papers (up to 5000 grit) and subsequently electrolytically polished. Vickers hardness measurements (ASTM E384 [23]) were performed on the RD–ND plane using an HVS-1000 microhardness tester (Jinan Hensgrand Instrument Co., Ltd., Jinan, China) with a load of 100 g and a dwell time of 10 s. For each loading-specified measurement, six data points were collected along the RD direction (to represent the average hardness at the same depth), as well as seven data points along the ND direction, forming a 6 × 7 rectangular grid with a spacing of 100 μm between adjacent indentations.

**Figure 1:**
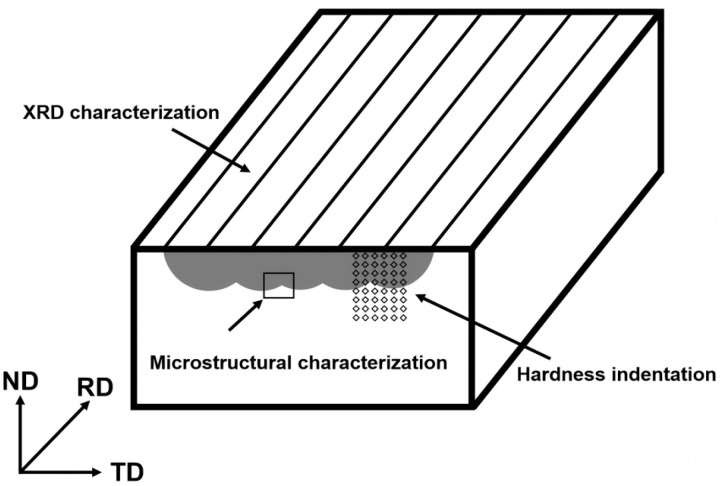
Schematic illustrating the conducted microstructural characterization and hardness testing.

A schematic illustrating the conducted microstructural characterization and hardness testing is presented in Figure 1.

## 3. Results and Discussion

### 3.1. Phase Constitution

**Figure 2:**
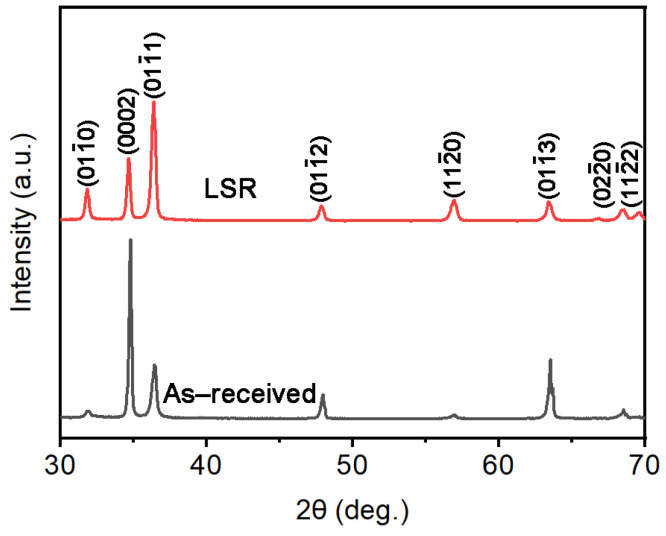
XRD patterns of the samples before and after LSR treatment.

Figure 2 shows the XRD patterns of the experimental samples before and after laser surface remelting (LSR). In both the samples before and after the LSR treatment, the main diffraction peaks correspond solely to the α-Zr phase with a hexagonal close-packed (HCP) structure. No diffraction peaks from second phase particles (SPPs) are observed, likely due to the low SPP content in this alloy [24]. A comparison of peak intensities reveals that the (0002) plane in the as-received sample exhibits a significantly stronger diffraction intensity than other planes. However, after LSR treatment, the intensity of the (0002) peak decreases markedly, while the intensities of the $\left(01\bar{1}0\right)$ and $\left(01\bar{1}1\right)$ planes increase considerably. This suggests that LSR processing may have induced changes in the crystallographic texture of the material.

### 3.2. Texture

**Figure 3:**
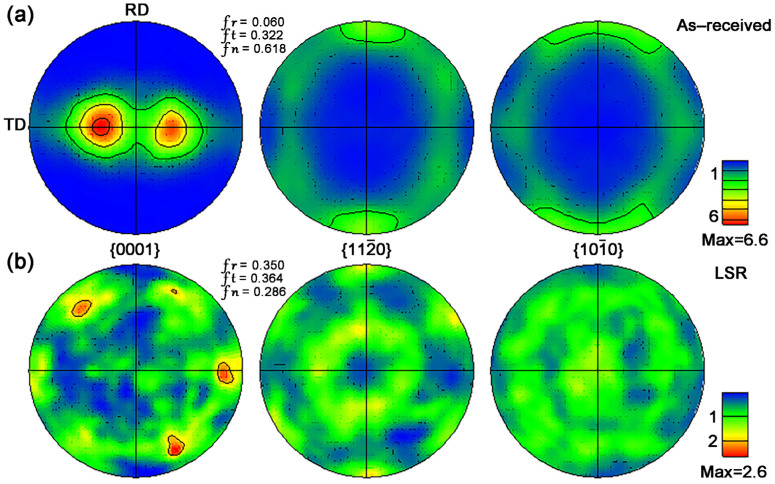
EBSD pole figures of the samples before and after LSR treatment (scanned area > 3 mm^2^): (**a**) as-received sample, (**b**) LSR-treated sample.

To further investigate the crystallographic texture characteristics, large-area EBSD measurements have been performed on both the as-received and LSR-treated samples, with pole figures shown in Figure 3. As illustrated in Figure 3a, the as-received sample exhibits a typical texture with bimodal basal features, with a maximum pole density of 6.6 [25]. In contrast, after laser surface treatment, the original texture feature completely disappears, and the maximum pole density drops to 2.6. Based on the calculated Kearns factors, the LSR-treated sample shows values much closer to a random texture (*f*_n_ = *f*_t_ = *f*_r_ = 0.333) [26], indicating a more disordered grain orientation near the treated surface.

During LSR, the surface of the zirconium alloy absorbs a significant amount of energy and experiences rapid melting, followed by solidification and a β → α phase transformation [19]. Typically, during the rapid β → α transformation in zirconium alloys, the orientation relationship follows the Burgers relationship. Under these conditions, a single β orientation can produce up to 12 distinct α variants, resulting in a more randomized crystallographic orientation within the transformed microstructure [27].

### 3.3. Microstructure

**Figure 4:**
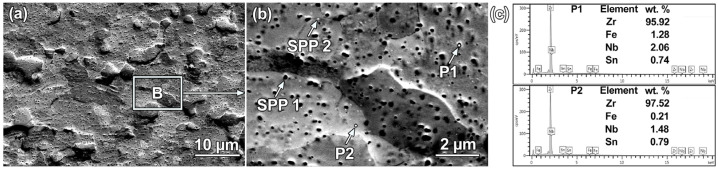
ECC imaging and EDS characterization of the as-received sample: (**a**) low-magnification ECC image; (**b**) enlarged view of the white-boxed area in (**a**); (**c**) EDS spectra acquired at points P1 and P2 in (**b**).

Figure 4 shows the electron channeling contrast (ECC) micrographs and corresponding EDS analyses of the as-received alloy. In the low-magnification ECC image (Figure 4a), the initial microstructure consists of fine equiaxed grains with an average size of 2.0 ± 0.6 μm. The boxed area in Figure 4a is magnified in Figure 4b, showing two types of randomly distributed nanoscale SPPs: the dark SPP-1 and the bright SPP-2 (indicated by arrows). EDS point analysis (Figure 4c) shows that the dark particles at P1 are enriched in Fe and Nb relative to the matrix at P2, whereas the SPP-2 particles are too small for reliable EDS quantification. Based on prior studies of this alloy system, the dark SPP-1 is attributed to the Zr(Nb,Fe)_2_ phase [28,29,30], while the finer bright SPP-2 should correspond to β-Nb [30,31].

**Figure 5:**
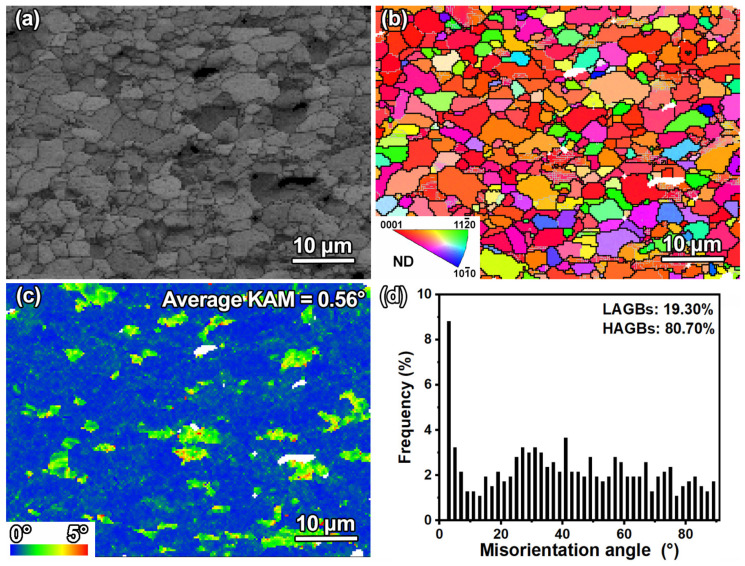
EBSD characterization of the as-received sample (step size = 0.3 μm): (**a**) BC map; (**b**) IPF map (black lines = HAGBs, gray lines = LAGBs); (**c**) KAM map; (**d**) misorientation angle distribution.

The EBSD results for the as-received sample are displayed in Figure 5. The band contrast (BC) map (Figure 5a) indicates a high-quality signal with uniformly distributed equiaxed grains, consistent with the observations in Figure 4a. The corresponding inverse pole figure (IPF) map in Figure 5b further reveals that most grain c-axes are oriented close to the ND direction, with grain boundaries predominantly consisting of high-angle grain boundaries (HAGBs). The misorientation distribution (Figure 5d) reveals that among all boundaries, HAGBs account for 80.70%, while low-angle grain boundaries (LAGBs) account only for 19.30%. The kernel average misorientation (KAM) map in Figure 5c shows an average KAM value of 0.56°, suggesting low residual strain within the grains [32].

**Figure 6:**
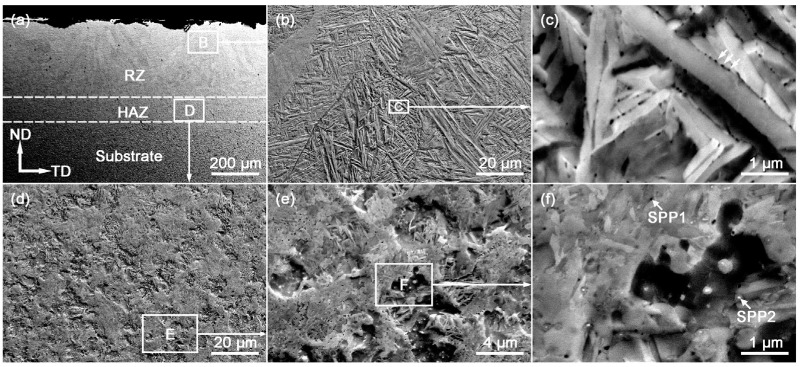
ECC imaging of the ND-TD cross-section of the LSR-treated sample: (**a**) low-magnification view; (**b**,**d**) higher-magnification observation of regions B and D in (**a**); (**c**,**e**,**f**) further magnifications of the boxed regions C, E, and F in (**b**,**d**,**e**).

The cross-section ECC images of the LSR sample (Figure 6) reveal three distinct regions: the unmodified substrate, a remelted zone (RZ) of approximately 350 μm thickness, and an adjacent heat-affected zone (HAZ) of about 70 μm thickness (roughly separated by dashed lines in Figure 6a). In the RZ (Figure 6b), the contours of former β-columnar grains remain visible. A further magnification of region C (Figure 6c) shows that the RZ comprises ultrafine α laths with black precipitates along the lath boundaries (white arrows). Using Nano Measurer software (Version 1.2.5), the average α lath width is measured to be 0.28 ± 0.10 μm, which is finer than reported α lath widths (0.32 and 1.2 μm) for LSRed Zr702 with respective laser fluences of 0.625 and 10 J·mm^−2^ [19]. During LSR, the near-surface region rapidly undergoes α → β → liquid transformation due to the high thermal input, followed by extreme undercooling during solidification. This promotes abundant nucleation of α laths and the precipitates along their interfaces [33,34,35]. The presence of dense α laths could also be expected according to the continuous cooling transformation (CCT) diagram of Zr alloys. For instance, Hunt and Niessen [36] figured out that cooling rates greater than ~500 °C/s allowed the nose of the CCT curve of Zr-Nb-O alloys to be well bypassed and promoted the nucleation and growth of α laths at the β boundaries. Since the cooling rate induced by the pulsed laser in the present work could exceed 1000 °C/s [19], dense fine α laths are easily produced.

In the HAZ (Figure 6d–f), the microstructure consists of retained blocky α grains, newly formed α laths, and precipitates, indicating an α + β → α transformation. Although the HAZ is heated into the α + β two-phase region, the temperature rise is insufficient for full melting; hence only some α grains transform via α → β → α to form laths, while the rest remain as blocky α grains upon cooling.

**Figure 7:**
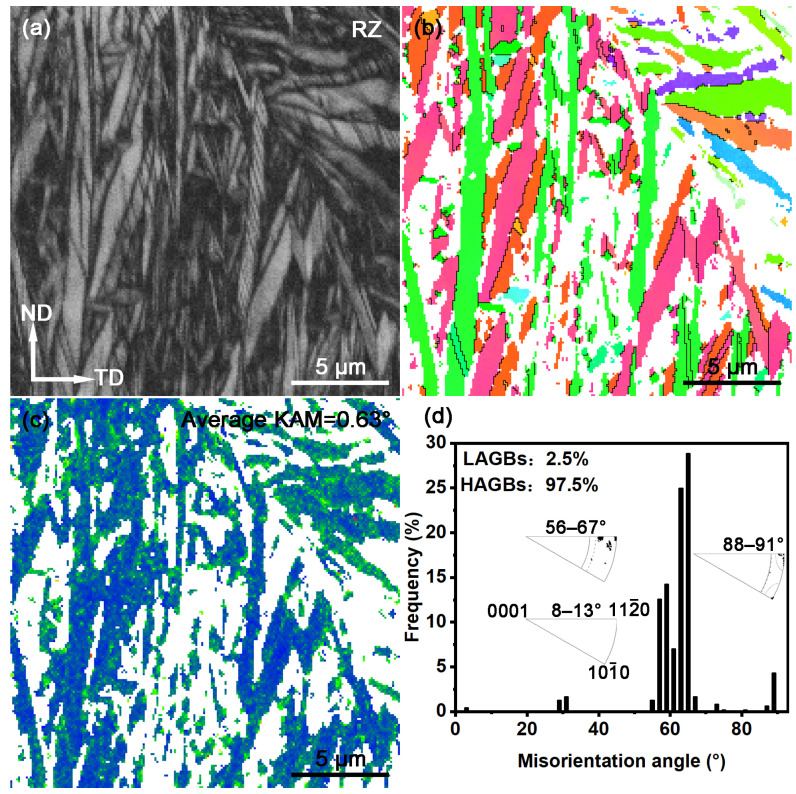
EBSD characterization of the RZ (step size = 0.1 μm): (**a**) BC map; (**b**) IPF map; (**c**) KAM map; (**d**) misorientation angle and rotation axis distribution. The Color code is the same as those in Figure 5.

The EBSD results for the remelted zone (RZ) of the LSR sample are presented in Figure 7. The BC map (Figure 7a) shows that the RZ is mainly composed of fine α laths, in agreement with the observations in Figure 6c. In general, the quality of a BC map is closely related to the defect density in the local area of the sample. Therefore, it can be inferred that the brighter regions in Figure 7a correspond to areas with lower defect density, while the darker regions (displayed as unindexed white areas in Figure 7b,c) indicate higher defect density. The IPF map in Figure 7b demonstrates that the grain orientations in the RZ are relatively scattered, with no pronounced texture. As shown in Figure 7c, the average kernel average misorientation (KAM) value in the RZ is 0.63°, slightly higher than the substrate (0.56°), indicating an increase in residual strain in the RZ after LSR. From the misorientation angle distribution shown in Figure 7d, it is evident that there is a significant concentration of misorientation angles around 60°, with some distribution near 90° as well. After analyzing their corresponding rotation axes (also shown in Figure 7d), it is clear that these angle/axis pairs are consistent with the features of the Burgers orientation relationship [37]. This suggests that during LSR the β → αtransformation in the RZ largely adheres to the Burgers orientation relationship (OR), resulting in the development of a relatively disordered crystallographic texture (as also seen in Figure 3b).

**Figure 8:**
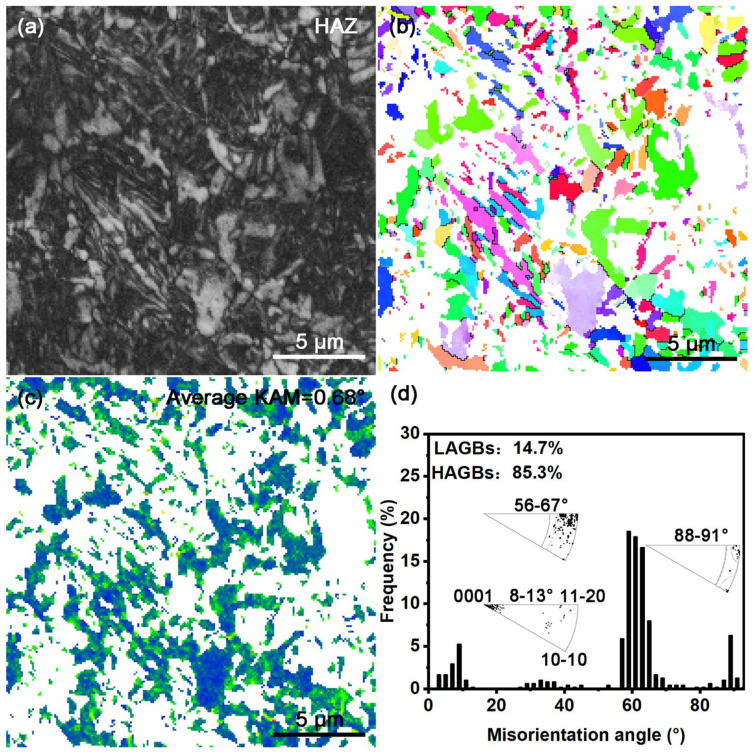
EBSD characterization of the HAZ (step size = 0.1 μm): (**a**) BC map; (**b**) IPF map; (**c**) KAM map; (**d**) misorientation angle and rotation axis distribution. The Color code is the same as those in Figure 5.

Figure 8 shows the EBSD results for the HAZ in the LSR sample. The morphological features in Figure 8a and b agree with the ECC observations in Figure 6e,f. Furthermore, the IPF map in Figure 8b indicates that the grain orientations in the HAZ are generally dispersed. The KAM map in Figure 8c shows that the average KAM value of the HAZ is 0.71°, which is slightly higher than those of the RZ (0.63°) and the substrate (0.56°), suggesting a higher level of residual strain in this region. In recent studies conducted for LSR-treated Zr702 and Ti-6Al-4V alloys, similar microstructural features were also found in their HAZs. More detailed analyses can be found in the relevant literature [19,38]. The rotation axis distribution and misorientation angle distribution shown in Figure 8d further demonstrate that the microstructure in the HAZ also well follows the Burgers OR during cooling.

### 3.4. Microhardness

**Figure 9:**
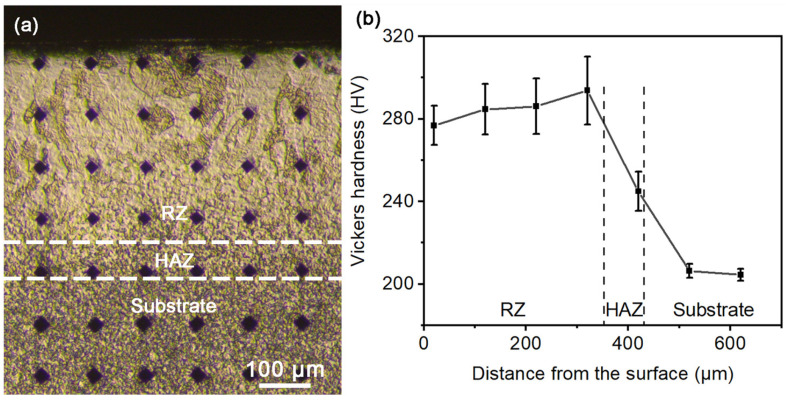
Vickers microhardness results of the TD-ND cross-section of the LSR-treated sample: (**a**) hardness indentation map; (**b**) variation of hardness with depth. The dashed lines roughly indicate interfaces between different zones.

Figure 9 presents the cross-sectional hardness measurements of the LSR-treated sample. In Figure 9a, the size of the Vickers indentations reflects the local hardness; it is clear that the indentation size in the RZ is markedly smaller than the substrate, reflecting a higher hardness in the RZ. This observation is confirmed by the depth-dependent hardness profile in Figure 9b, which shows that both the RZ and HAZ exhibit higher hardness than the substrate after LSR treatment. The average hardness of the RZ is measured to be 285.7 ± 8.3 HV, representing an increase of ~40% compared to the substrate hardness (204.5 ± 5.6 HV). This marked improvement is mainly attributed to the LSR-induced grain refinement, which increases grain boundary density and thereby enhances resistance to dislocation motion [19].

According to the Hall–Petch equation, the hardening contribution from grain refinement can be expressed as follows, $\Delta HV=k_{HP}^{H}(d_{LSR}^{-0.5}-d_{AR}^{-0.5})$, where ΔHV is the hardness increment, $k_{HP}^{H}$ is the Hall–Petch coefficient (in units of Vickers hardness), $d_{AR}$ is the average grain size of the substrate, and $d_{LSR}$ is the average width of α laths in the RZ. Using a Hall–Petch coefficient ($k_{HP}^{H}$ = 66.7 HV μm^1/2^) of α-Zr [18], the ΔHV can be calculated to be 78.9 HV, close to the measured hardness increment (285.7 HV − 204.5 HV = 81.2 HV). This suggests that LSR-induced grain refinement should have made the major contribution to the surface hardening of the zirconium alloy. Nevertheless, as shown in Figure 6c, a few fine precipitates are distributed along the α lath boundaries in the RZ, which may further hinder dislocation movement and contribute to the hardness enhancement. In addition, for zirconium alloys with an HCP structure, mechanical properties such as strength and hardness are also closely correlated with crystallographic orientation [39]. For instance, Yang et al. [40] reported that the hardness of α-Zr grains was closely related to the angle φ between the grain’s c axis and the loading direction; as φ increases (up to a maximum of 90°), the hardness tends to decrease. In this study, the hardness test was performed along the RD direction. According to Figure 3, most grains in the substrate have a φ value close to 90°, corresponding to a soft orientation. In contrast, the RZ of the LSR-treated sample exhibits a random texture, implying a random distribution of φ values in the range of 0–90°, with both soft- and hard-oriented grains evenly distributed. This random texture may also have mild contribution to the higher hardness observed in the RZ. Meanwhile, the scattered grain orientations in the RZ should also be responsible for its larger hardness deviations than the substrate.

Regarding the HAZ, it has undergone an α + β → α phase transformation, resulting in the formation of fine α laths (as seen in Figure 6f and Figure 8a), which induces a certain degree of grain refinement. However, since part of the residual α grains does not undergo phase transformation, the hardness enhancement in the HAZ is less pronounced, with an average value of approximately 240 HV (markedly lower than the RZ’s hardness). The more scattered hardness of the HAZ than the substrate could be attributed to its less homogeneous microstructure produced by the α + β → α transformation.

## 4. Conclusions

(1) After the LSR treatment, two distinct microstructural regions are formed on the surface of the Zr-1.0Sn-1.0Nb-0.3Fe zirconium alloy: (*i*) the RZ—consisting of fine α lath structures and precipitates distributed along the lath boundaries—and (*ii*) the HAZ—composed of blocky α grains, α laths, and precipitates.
(2) The initial sample exhibits a typical bimodal basal texture, while the LSR-treated sample surface shows a random texture. This change can be attributed to the selection suppression of α variants during laser-induced rapid cooling.
(3) Following the LSR treatment, the average hardness of the RZ rises to 285.7 ± 8.3 HV, ~40% higher than the substrate. This enhancement is primarily ascribed to the effective grain refinement induced by LSR.

Considering the practical applications of zirconium alloys as nuclear components (e.g., fuel claddings), enhanced surface hardness is very beneficial for improving their relatively limited wear/fretting resistance to ensure an extended service life. In addition to the demonstrated effectiveness of surface hardening, more comprehensive property evaluations are to be conducted for LSR-induced zirconium alloys.

## Figures and Tables

**Table 1:** Chemical composition of the experimental material (wt.%).

Element	Nb	Sn	Fe	O	Zr
Content	1.0	1.0	0.3	0.12	Bal.

**Table 2:** Processing parameters for pulsed laser surface remelting.

Power (W)	Peak Power (kW)	Laser Fluence (J·mm^−2^)	Frequency (Hz)	Pulse Width (ms)	Defocus Distance (mm)	Scanning Speed (mm·s^−1^)	Laser Spot Diameter (mm)
50	0.5	1.25	20	5	+2	8	1

## Data Availability

The original contributions presented in this study are included in the article. Further inquiries can be directed to the corresponding authors.
